# The Omics Complexity in Sepsis: The Limits of the Personalized Medicine Approach

**DOI:** 10.3390/jpm14030225

**Published:** 2024-02-20

**Authors:** Sebastian Isac, Teodora Isac, Maria Daniela Tanasescu, Bogdan Pavel, Cristina Veronica Andreescu, Andrada-Georgiana Badea, Damiana Ojog, Geani-Danut Teodorescu, Anca Laceanu, Cristian-Bogdan Trifan, Gabriela Droc

**Affiliations:** 1 Department of Physiology, Faculty of Medicine, Carol Davila University of Medicine and Pharmacy, 020021 Bucharest, Romania; bogdan.pavel@umfcd.ro; 2 Department of Anesthesiology and Intensive Care I, Fundeni Clinical Institute, 022328 Bucharest, Romania; andrada-georgiana.badea0721@stud.umfcd.ro (A.-G.B.); damiana.p.ojog@stud.umfcd.ro (D.O.); geani-danut.teodorescu0721@stud.umfcd.ro (G.-D.T.); anca.laceanu@stud.umfcd.ro (A.L.); cristian.trifan@stud.umfcd.ro (C.-B.T.); gabriela.droc@umfcd.ro (G.D.); 3 Department of Internal Medicine II, Faculty of Medicine, Carol Davila University of Medicine and Pharmacy, 020021 Bucharest, Romania; teodora.isac@drd.umfcd.ro; 4 Department of Medical Semiology, Discipline of Internal Medicine I and Nephrology, Faculty of Medicine, Carol Davila University of Medicine and Pharmacy, 020021 Bucharest, Romania; maria.tanasescu@umfcd.ro; 5 Department of Modern Languages, Faculty of Medicine, Carol Davila University of Medicine and Pharmacy, 020021 Bucharest, Romania; cristina.andreescu@umfcd.ro; 6 Department of Anesthesiology and Intensive Care, Fundeni Clinical Institute, Faculty of Medicine, Carol Davila University of Medicine and Pharmacy, 020021 Bucharest, Romania

**Keywords:** sepsis, septic shock, cytomics, genomics, epigenomics, transcriptomics, proteomics, metabolomics, clinical phenotypes

## Abstract

Sepsis is one of the most common causes of morbidity and mortality worldwide. Despite the remarkable advances in modern medicine throughout the last century, the mortality rates associated with sepsis have remained significantly elevated, both in high- and low-income countries. The main difficulty in the diagnosis and treatment of septic patients is the tremendous heterogeneity of this condition. The vast heterogeneity that characterizes sepsis ranges from the clinical presentation to the biological aspects of the disease. Evidence-based medicine approaches sepsis as a homogenous syndrome and does not consider the individual discrepancies between septic patients. This approach may contribute to the poor outcomes of septic patients. In recent years, personalized medicine has gained significant interest. This novel form of medicine underlines the importance of understanding the genetic, epigenetic, and molecular basis of a disease in order to provide a more tailored approach for the patient. The study of “omics”, such as cytomics, genomics, epigenomics, transcriptomics, proteomics, and metabolomics, provides a deeper comprehension of the complex interactions between the host, the disease, and the environment. The aim of this review is to summarize the potential role of a personalized approach in sepsis management, considering the interactions between various “omics”.

## 1. Introduction

The definitions of sepsis and septic shock have varied greatly over the years. Sepsis is a clinical syndrome defined as life-threatening organ dysfunction caused by a dysregulated host immune response to infection. Septic shock is defined as a subset of sepsis characterized by circulatory, cellular, and metabolic abnormalities and associated with a higher risk of mortality than sepsis [1].

Sepsis and septic shock constitute major healthcare problems. The global reported incidence of sepsis is 19.4 million cases annually, but this could be even greater considering the data scarcity in low-income countries [2].

The mortality associated with sepsis and septic shock is still high despite the constant improvement of diagnostic and therapeutic strategies. The mortality rates range from 15% to 56%, respectively [3]. 

**Figure 1:**
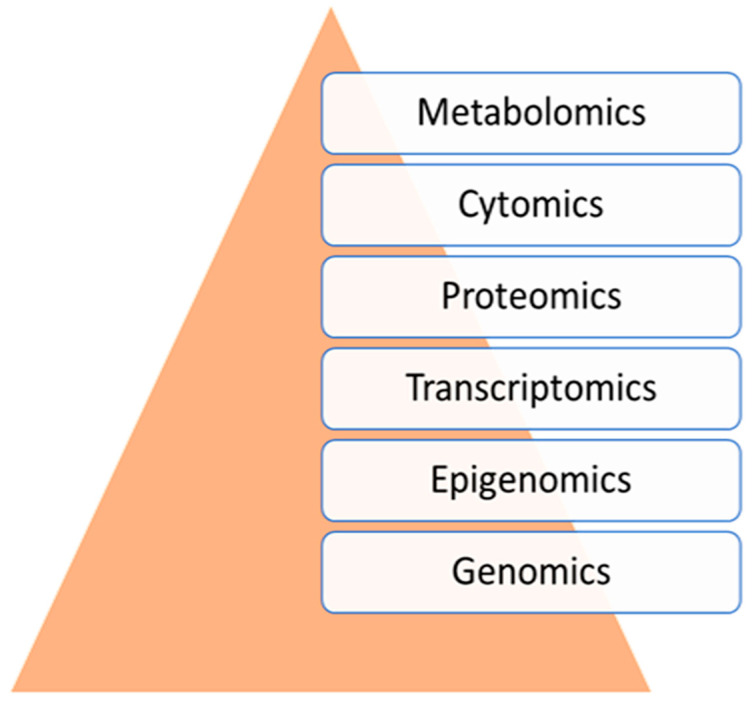
Heterogeneity sources in sepsis.

The latest diagnostic criteria for sepsis rely on the Sequential Organ Failure Assessment (SOFA) score, the presence of infection, the lactate level in the plasma, and the mean arterial pressure. Additionally, a mean arterial pressure under 65 mmHg in the presence of sepsis defines septic shock [1]. Thus, according to the definition and diagnostic criteria, sepsis is a complex clinical entity [4,5]. One of the main aspects that contributes to the heterogeneity of sepsis is the dysregulated host immune response to infection and various interactions between the host genome and the infectious trigger [6]. Despite the modern clinical tools for the prediction of sepsis, machine learning systems (i.e., artificial intelligence) could significantly reduce the presentation to diagnosis time interval, improving the outcomes of septic patients [7]. By counteracting the variable immune response, artificial intelligence might consider the various levels of heterogeneity and its sources in sepsis (Figure 1). 

The aim of this review is to reveal the complexity of the various mechanisms in sepsis, considering the study of omics, as the main source of its heterogeneity. 

## 2. Cytomics

Cytomics refers to the study of immune cells’ responses to various sepsis-specific activators and assesses, from this perspective, the evolution of the disease.

**Figure 2:**
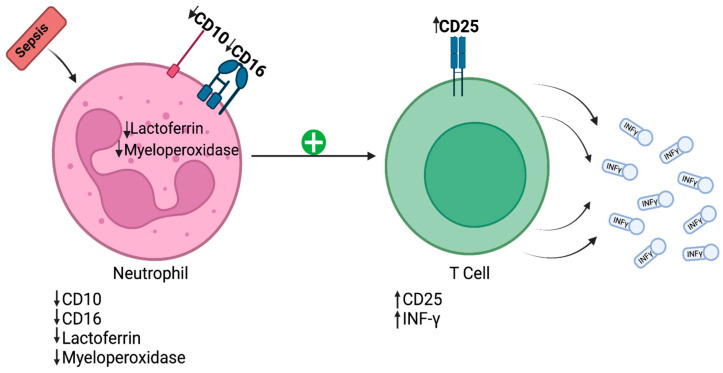
Initial immune response to infectious injury in sepsis. CD—cluster of differentiation; IFN-γ—interferon-gamma.

Different studies of immune cells have revealed certain changes in sepsis patients’ neutrophils. Toxic granulation within neutrophils and the vacuolization of neutrophil cytoplasm represent the initial immune response to infectious injury in sepsis [8]. Furthermore, the structural complexity of this response is related to its magnitude. The interaction between neutrophils and T lymphocytes in sepsis is revealed in Figure 2.

Recently, a class of granulocytes named “low-density neutrophils” (LDN) has been described [9]. According to Ran Sun et al., LDN formation is closely related to neutrophil degranulation, and these LDN provide a lower level of immune defense in sepsis compared to high-density neutrophils [9]. Aside from exhibiting a reduced phagocytic capacity and lower chemotaxis, these cells also have a longer lifespan than regular neutrophils [9]. In addition, these cells express CD15 on their surfaces and suppress T cell proliferation.

Additionally, monocytes lacking the human leucocyte antigen-DR isotype (HLA-DR) were deficient in terms of their antigen-presenting capacity and in producing TNF-α (inflammatory cytokine). T lymphocytes also have been described to overexpress inhibitory markers such as T cell immunoglobulin and mucin-domain-containing protein 1 (TIM-1), lymphocyte activation gene 3 (LAG-3), and cytotoxic T-lymphocyte-associated protein 4 (CTLA4). T cell hyperactivation was also noticeable in afflicted patients: either through antigen-dependent activation or through an antigen-independent “bystander” activation [10]. 

Furthermore, the reduced expression of leukocyte-related antigen D (mHLA-DR) influences the evolution of ICU patients. Based on patients’ mHLA-DR expression variations compared to the mHLA-DR reference interval (13,000–42,000 AB/C—number of antibodies bound per monocyte), Maxime Bodinier et al. divided patients into four endotypes: “non-improvers”, “decliners”, “improvers”, and “high expressors” [11]. “Non-improver” is an endotype displaying constant expression below the mean (<4000 AB/C), while the expression in “decliners” starts at the reference values and decreases below 7500 AB/C. The “improver” endotype is characterized by an increase in mHLA-DR expression, almost reaching the reference interval, at the end of the first week. The endotype with mHLA-DR rapidly reaching the reference interval is referred to as “high expressor”. The “non-improver” and “decliner” endotypes were associated with worse outcomes: a longer length of ICU stay and exposure to invasive devices, and increased death and ICU-acquired infections, compared to the “improver” and “high expressor” endotypes [11].

Moreover, Arjun Baghela et al. showed that the progression of sepsis in certain patients was influenced by specific cellular characteristics, such as the prevalence of signaling pathways and genetic expression [12]. The neutrophilic-suppressive (NPS) endotype is associated with the activation of neutrophils, immune suppression, and the interleukin 6–signal transducer and activator of transcription (IL6/STAT) unique pathway. Studies have shown its progression to be the most severe, with the highest Sequential Organ Failure Assessment (SOFA) scores and the worst survival rate. The NPS endotype involved upregulated genes in neutrophil degranulation, NOD-like receptor (NLR) pathways, and reactive oxygen species production. It also showed the downregulation of adaptive immune and interferon signaling pathways [12].

Thus, various subcellular endotypes, reveled through cytomics study, could finally dictate the outcome in sepsis and septic shock.

## 3. Genomics

Genomics studies various relevant gene polymorphisms that could influence the probability of developing sepsis and septic shock and the treatment response. 

Toll-like receptors (TLR) are molecules found in the membranes of various immune cells and play a crucial role in initiating the innate immune response. TLRs recognize pathogen-associated molecular patterns (PAMPs) present on bacteria, fungi, protozoa, and viruses. These PAMPs bind to TLRs and initiate the transcription of genes that code for various pro-inflammatory cytokines, chemokines, and antibody production. The main activation mechanisms rely on the NF-kB pathway. Several studies have shown that mutations in the TLR genes can lead to higher susceptibility to sepsis and septic shock [13,14,15].

**Figure 3:**
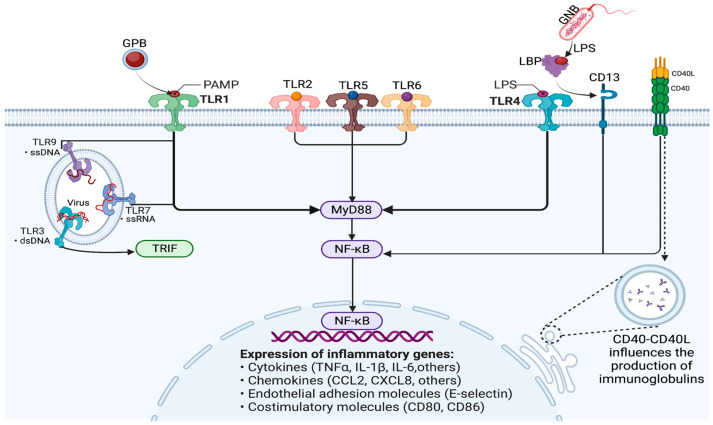
Modulation of immune response and gene expression through the NF-kB signaling pathway in sepsis. GPB—Gram-positive bacteria; GNB—Gram-negative bacteria; PAMP—pathogen-associated molecular pattern; TLR—Toll-like receptor; LPS—lipopolysaccharide; LBP—lipopolysaccharide-binding protein; NF-kB—nuclear factor kappa B; MyD88—myeloid differentiation primary response 88; TRIF-TIR—Toll/interleukin-1 receptor domain-containing adaptor protein inducing interferon beta, also known as TICAM-1 (TIR-containing adaptor molecule 1); ssDNA—single-stranded DNA; dsDNA—double-stranded DNA; ssRNA—single-stranded RNA; CD—cluster of differentiation; CD40L—CD40 ligand; TNF-α—tumor necrosis factor alpha; IL—interleukin; CCL2—chemokine (C-C motif) ligand 2; CXCL2—C-X-C motif chemokine ligand 8.

NF-kB is expressed in almost all cells of the body and normally exists in the cytosol, bound to an inhibitor of the IkB family [16]. The main NF-kB-dependent mechanisms in sepsis are revealed in Figure 3.

CD40 is a receptor protein expressed by various immune cells. The CD40 ligand (CD40L) binds to the CD40 receptor on B cells and dendritic cells and thus influences the production of immunoglobulins and, consequently, the probability of developing sepsis [17]. 

Variability in the genes responsible for the NF-kB pathway can influence mortality and morbidity in septic shock. In a retrospective study, genotyping single-nucleotide polymorphisms in a couple of genes allowed the definition of the CC genotype of NIK rs7222094, which was demonstrated to be associated with increased mortality and more renal and hematological failure than the CT and TT genotypes (T—mutated allele, C—normal allele). Moreover, an important chemokine, CXCL10, released by activating NF-kB, had lower levels in the CC genotype in septic shock patients. Thus, polymorphisms in the NF-kB genes could predict the risk of death or the response to target therapies [18].

A recent study led by Sun J. et al. aimed to illustrate the correlation between single-nucleotide polymorphisms (SNPs) of the NFKB1 gene and sepsis-associated acute kidney injury (AKI) [19]. The result showed that the carrier group of SNPs (A-allele carriers) on the rs41275743 and rs4648143 loci of the NFKB1 gene was associated with a risk of developing AKI 1.46 that was 1.56 times higher than the risk of the cluster lacking SNPs (G-allele carriers). Moreover, Thair et al. studied the influence of NF-kB genetic variation on the outcome of septic shock [20]. The results revealed that a single-nucleotide polymorphism (SNP) in the NF-kB-inducing kinase was significantly associated with increased chances of renal and hematological dysfunction and with greater 28-day mortality. The degree of NF-kB activation is also linked to illness severity [21]. On the other hand, it was also shown that a deficit in the activation of NF-kB in sepsis is a factor involved in inducing complications (acute lung injury) and increasing the illness severity [22].

**Figure 4:**
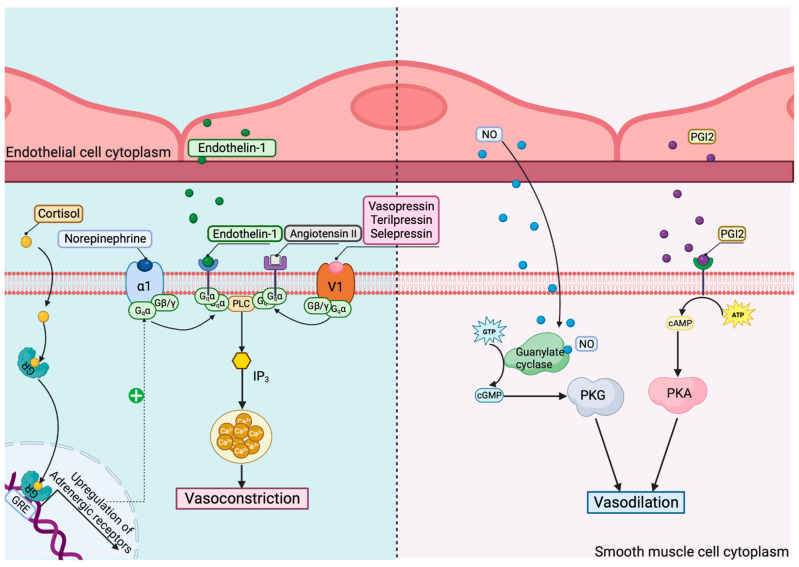
Mechanisms and pharmacological modulation of vasomotor dysregulation in septic shock. GR—glucocorticoid receptor; GRE—glucocorticoid response elements; V1—vasopressin receptor 1; α1—α1 adrenoreceptor; PLC—phospholipase C; IP3—inositol trisphosphate; NO—nitric oxide; PGI2—prostaglandin I2; PKG—protein kinase G; PKA—protein kinase A.

In septic shock, there are several mechanisms that affect the vasomotor tonus of blood vessels, leading to systemic hypotension. The main mechanisms and their pharmacological modulation are revealed in Figure 4. 

Consequently, a differentiated genomic-targeted approach for vasopressor therapy in septic shock should be applied. Norepinephrine is the first choice in septic shock [1]. In some cases, such as septic patients with catecholamine-refractory shock, the treatment of choice should be reconsidered, with some alternatives being the catecholamine-sparing agent arginine vasopressin (AVP), corticosteroids, and angiotensin II. Terlipressin (TP), the long-lasting analog of vasopressin, has higher affinity for V1 receptors, which mediate vasoconstriction; thus, it might be considered a more optimized therapeutic option. Selepressin is also an agonist for vasopressin and an effective substitute for norepinephrine. Angiotensin II, a critical regulator of the blood volume and systemic vascular resistance, has proven efficient when administered together with norepinephrine, because it decreases the dosage of catecholamine necessary while avoiding its unwanted effects [23,24]. 

An important risk factor in norepinephrine- as well as vasopressin-treated patients is the presence of single-nucleotide polymorphisms (SNPs) that affect their receptors. Thus, a genomic-based approach involving key receptor polymorphisms might influence the therapeutic strategy and outcome in patients with refractory septic shock. Nakada et al. studied the influence of ADRB2 (gene for β2-adrenergic receptor) polymorphisms on the outcomes of septic shock treated with norepinephrine [25]. Their study revealed that a certain ADRB2 gene polymorphism was associated with greater cardiovascular dysfunction, organ dysfunction, and mortality in patients with septic shock. They described three frequent single-nucleotide polymorphisms: Cys/Arg-19, associated with altered gene translation in vitro, and Gly/Arg16 and Gln/Glu27, responsible for receptor expression in vitro and receptor desensitization in vivo. Homozygotes for this Cys/Gly/Gln haplotype are known as the AA genotype of the ADRB2 rs104271 and this was correlated with increased 28-day mortality, more organ failure, a higher heart rate in the first 5 days, and higher norepinephrine administration in septic patients (decreased receptor expression, decreased anti-inflammatory effect) [25].

According to the VASST study, the autosomal dominant form of rs28418396 SNP is a risk factor for serious adverse events (SAEs; associated with the use of vasoconstrictor therapy) [26]. The identified SNP is located near the 5’-UTR of the gene responsible for coding arginine vasopressin receptor 1B (AVPR1b), whose stimulation releases stress hormones, like cortisone. Additionally, a higher SAE probability was identified in patients with the AA genotype of rs28418396 compared to those with the TT/TA genotype. 

Aside from this mechanism, glucocorticoids impact the vascular tone and have a pivotal role in septic shock [27]. According to De Kloet et al, SNPs in the glucocorticoid receptor (GR) impact the metabolic profile and cardiovascular parameters: ER22/23EK, with a favorable profile (decreased the response to cortisol), and N363S together with Bcl1, with a less advantageous one (they increased the cortisol responses to various types of stress, causing immunosuppression in the patient and a predisposition to infection) [28]. Once sepsis is induced, the immunosuppressive effect of glucocorticoids is beneficial, reducing the transcription of pro-inflammatory genes via the inhibition of nuclear factor kappa B [27]. The hGR NS-1 form, described by Baker et al., contains three nonsynonymous SNPs (A214G, T962C, and A2297G) [29]. The presence of either SNP A214G or T962C resulted in a decreased response to dexamethasone in sepsis. However, if methylprednisolone was administered, the presence of SNP A214G resulted in greater activity when compared with hGR, whereas T962C resulted in activity equivalent to hGR [29].

Thus, the interindividual variability of the mechanisms that modulate the NF-kB-mediated immune response and catecholamine-responsive vascular tone, revealed by genomic studies, could contribute to personalized medicine approaches in sepsis and septic shock. 

## 4. Epigenomics

**Figure 5:**
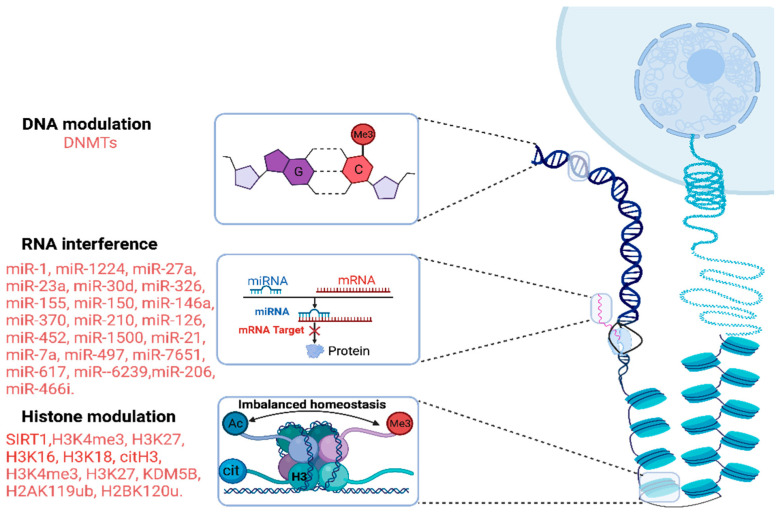
Epigenetic markers and the main miRs present in sepsis. G—guanine; C—cysteine; Me—methyl group; DNMTs—DNA methyltransferases; Ac—acetyl group; mRNA—messenger RNA; miR—microRNA; SIRT1—silent mating type information regulation 2 homolog 1; citH3—citrullinated histone H3.

Epigenomics is a rapidly evolving field of study that explores how the environment and other external factors can influence gene expression and impact human health, without modifying the gene structure and sequence [30]. Epigenetic mechanisms could explain the expression heterogeneity of genes involved in the immune response and inflammation, which are key factors in the development and progression of sepsis [31]. The main epigenetic markers in sepsis are revealed in Figure 5.

Furthermore, targeted epigenetic therapies could offer a new perspective in a future individualized approach for septic patients. Considering the systemic impairments secondary to sepsis, various epigenetic tools are debated, nowadays, in the literature based on their organ specificity. 

Various epigenetic markers like microRNAs (mi-R) have been revealed as potential modulatory mechanisms in sepsis. According to Gao et al., miR-1-3p altered the cellular interaction in the lung, through exosomal mechanisms, in an experimental model of acute lung injury [32]. Specifically, high levels of miR-1-3p impaired the growth ability of the cells, reduced cell viability, and increased fluid leakage. Conversely, miR-27a is an alleviating factor in ALI by preventing TLR4/MyD88/NF-κB activation [33]. 

The levels of miR-23a-5p were found to be elevated in lipopolysaccharide (LPS)-treated mice and it has been shown that the pharmacological inhibition of this miRNA remarkably ameliorates the pulmonary injury and dysfunction induced by LPS [33]. Furthermore, miR-30d-5p released through exosomes by PMNs is a key component in macrophage-mediated tissue damage in the lungs [34]. miR-30d-5p promotes M1 macrophage polarization and pyroptosis. miR-326 downregulates TLR4 in macrophages, therefore diminishing ALI [35]. miR-155 is responsible for many aspects of immune cell regulation and is closely related to inflammatory and autoimmune conditions. As a biomarker of sepsis, it has been confirmed to be upregulated in sepsis patients [36]. In addition, it has been shown that the reduction of miR-155 prevents acute lung injury in the setting of sepsis [37].

Moreover, Jiao et al. suggested that the interaction between polymorphonuclear neutrophils and macrophages in sepsis could also be epigenetically modulated [34]. miR-30d-5p plays a key role in sepsis-induced macrophages’ polarity and kinesis, leading to inflammation and secondary lung damage. This marker is expressed by the PMN exosomes in a complex intercellular interaction [34].

miR-1224-5p inactivates the PPAR-γ/AMPKα axis, which further impairs energy metabolism and peripheral insulin resistance, prevalent in septic patients.

Sepsis-induced encephalopathy (SIE) could also be epigenetically targeted. The current literature highlights the key role of miR-146a-5p in neuroinflammation, including SIE [38]. SIE is characterized by inflammation and various brain impairments. According to Zou et al., sepsis causes the upregulation of miR-146a-5p in mice and humans, targeting the Toll-like receptor 7 (TLR7) protein [38]. miR-370 is the most described biomarker in relation to SIE [39]. It is known to be highly specific for SIE, as its levels are undetectable in patients with other inflammatory diseases or sepsis. From a therapeutic perspective, miR-210 is an interesting candidate for the development of a miRNA-based therapy, as it exhibits neuroprotective effects, by inhibiting apoptosis [40]. This molecule is upregulated in a hypoxemic state, as in the ischemia–reperfusion phenomenon associated with SIE. Finally, miR-155 might also play a role in neuroinflammation, and its downregulation in microglia was shown to induce endotoxin tolerance [41]. 

Endothelial leakage is one of the most important features in sepsis [42]. Formosa et al. suggest that the levels of miR-150 could be an important epigenetic biomarker for the identification of patients at risk for endothelial dysfunction [43]. miR-150 suppresses Ang2 (modulates angiotensin) levels, which prevents the impairment of adherent junctions reannealing after the injury [44]. Moreover, miR-150 levels can be a marker of early sepsis and may be correlated with the severity of sepsis [36]. Etzrodt et al. revealed that miR-155 was upregulated in the endothelium during the systemic inflammatory response and it caused increased vascular permeability [45]. According to Zhou et al., high levels of miR-126 could play a protective role through the inhibition of LPS-induced vascular cell adhesion molecule 1 (VCAM1) levels and high-mobility box group 1 (HMBG1) levels [46]. 

Sepsis-induced acute kidney injury (AKI) has a complex pathophysiology involving interactions between immune cells, endothelial cells, tubular epithelial cells, and inflammatory mediators. 

Recent evidence indicates that epigenomic modifications play a crucial role in the development and progression of AKI secondary to sepsis. Liu et al. have found that urinary miR-452 could be an effective diagnostic marker of AKI in sepsis patients [47]. miR-1500 might also be a potential novel biomarker for the diagnosis of AKI, but it may also act as a therapeutic target. 

A molecule of potential therapeutic importance is miR-21. Zhang et al. found that miR-21-5p elevation through endothelial progenitor cell exosomes improved renal function and decreased the serum inflammatory response [48]. 

Myocardial injury in sepsis can manifest as myocardial depression, a decreased cardiac output, and the development of myocardial-infarction-like symptoms [42]. 

Liang et al. revealed that miR-7a-5p downregulation decreased apoptosis in mice cardiomyocytes in response to LPS [49]. miR-150 could also be involved in LPS-induced apoptosis [50]. A molecule of diagnostic interest is miR-497, which was found to have specificity of 91.2% and sensitivity of 90.4% for myocardial injury in children, being of similar value to cTnI, making it a potential biomarker for the early diagnosis of cardiac injury [51].

The immune response in sepsis that causes a cytokine storm is of paramount importance. Based on various endotypes, the sepsis-related immune response could be either overactive or suppressed, depending on various factors and pathogens [52]. One way to modulate an exacerbated immune response is to use exosome therapy. Tumor cells, which are known for their ability to suppress the immune system, also secrete exosomes that can be used to help to control the immune response in sepsis [53]. Moreover, miR-7651-5p, miR-615-5p, miR-6239, miR-690, miR-206-3p, miR-466i-5p, and miR-146a-5p, which are found in tumor-derived exosomes, may be responsible for their beneficial effects in sepsis. These microRNAs can be packaged into exosome mimics, which are designed to be more stable and effective in delivering the microRNAs to their target cells [54]. These exosome mimics may be a promising new therapy for sepsis. Likewise, the pre-existing miR background in the immune cells could serve as a potential prognostic tool by differentiating septic patients of various epigenotypes. Furthermore, specific epigenotypes could promote individualized genetic counselling and therapeutic strategies in sepsis.

Besides miRNAs, histone alterations, such as methylation, ubiquitination, acetylation, lactylation, and citrullination, are involved in the pathology of sepsis. This leads to delayed wound healing and long-term inflammatory damage in sepsis [55]. A potential mechanism is the sepsis-induced downregulation of various H3K4 and H3K27 methyltransferases, which contributes to the dysregulation of the histone methylation pattern. Sepsis disrupts also the homeostasis between histone acetylation and deacetylation [56]. Sirtuin 1 (SIRT1), a deacetylase, has received widespread recognition in sepsis [57]. SIRT1 acts in the IL-6 and TNF-α promoter regions by reducing H3K16 acetylation, blocking their expression [58,59]. Citrullination is a phenomenon of growing interest, as circulating citrullinated histone H3 (CitH3) has been shown to be a potential diagnostic marker for early sepsis, also reflecting the severity of sepsis [60]. Another biomarker of diagnostic and prediction value is lactylate histone H3K18 (histone 3 at lysine placed on position 18), which might play a role in inflammatory cytokine expression [61]. The mono-ubiquitination of core histones also contributes to altering the DNA. The mono-ubiquitination of H2AK119 and H2BK120 reduces the levels of IL-6 and IL-23a via the Nf-kB pathway. 

Finally, epigenetic DNA changes could impact the magnitude of the immune response in septic patients. The activity of DNA methyltransferases (DNMT) is also a noteworthy factor in the progression of sepsis. According to Luxi Cao et al., extracellular circulating DNMTs might be a predicting factor for sepsis severity. Their study on mice post-CLP showed the significant upregulation of DNMT1 (involved in copying methylation patterns during DNA replication). They theorized that Decitabine, a DNMT inhibitor, might prevent the extensive activation of the NF-kB pathway; however, further studies are needed to establish the necessary doses and timing [62]. 

Thus, epigenomics could be helpful in future personalized approaches in septic patients [62]. 

## 5. Transcriptomics

Transcriptomics, which implies the analysis of all the RNA transcripts present in a cell, has developed into an important vehicle for the comprehension of the intricate mechanisms underlying sepsis [63]. Thus, transcriptomics is responsible for identifying various patient profiles, based on RNA expression and protein synthesis, which are associated with different outcomes. This particular mechanism of gene expression could have an important prognostic and therapeutic role in sepsis and septic shock. 

The transcriptomic analysis of peripheral blood leukocytes in patients with sepsis caused by community-acquired pneumonia identified two groups of patients based on the sepsis response signatures: SRS1, a suppressed immune system phenotype, and SRS 2, an unsuppressed immune system phenotype [64]. The SRS1 phenotype was characterized by endotoxin tolerance, early T cell exhaustion, the downregulation of HLA class II proteins, and higher 14-day mortality. In contrast, the SRS2 phenotype was characterized by the absence of these deficiencies, being capable of an enhanced cellular response to antigens [64].

In Ref. [64], additionally, Burnham et al. compared the transcriptomic signatures of septic patients admitted to the intensive care unit for fecal peritonitis with those admitted for community-acquired pneumonia [65]. After analyzing the RNA from blood leukocytes, two groups were described among the fecal peritonitis patients, SRS1_FP and SRS2_FP, which were compared with the SRS1 and SRS2 phenotypes. Genes involved in endotoxin tolerance, T cell activation, cell death, apoptosis, and necrosis were upregulated in the SRS1 group, which was associated with higher early mortality. The findings showed that 46% of patients switched their SRS phenotype (the majority from SRS1 to SRS2) in the first 5 days, suggesting that the transcriptomic response signature is a highly sensitive predictive tool if considered as early as possible after patients’ admission [65].

A prospective cohort study revealed four molecular endotypes for sepsis, depending on the expression of 140 genes, and attempted to establish biomarkers for each one, as follows: Mars 1 (BPGM, TAP2), Mars 2 (GADD45A, PCGF5), Mars 3 (AHNAK, PDCD10), and Mars 4 (IFIT5, GLTSCR2) [66]. Patients in the Mars 1 group had the worst outcomes, with increased mortality. The poor prognosis in the Mars 1 endotype was due to a decline in the activity of genes related to the innate and adaptive immune system, such as the T cell receptor pathway and the NF-kB pathway, and the upregulation of cell metabolic activity (specifically heme biosynthesis), with these changes resembling immune exhaustion. Individuals in Mars 2 and Mars 4 exhibited an increase in pattern recognition and cytokine pathways, indicating a state of hyperinflammation. Mars 3, characterized by the enhanced activity of adaptive immunity (B cell development, IL-4 signaling), was correlated with the lowest risk [66]. 

Another study that combined data from 14 transcriptomic datasets described three sepsis subtypes in patients with bacterial sepsis: inflammopathic, adaptive, and coagulopatic. The inflammopathic group had an overactive innate immune system (an excessively defined inflammation-associated pathway through IL-1 receptors and the complement activation pathway) and a partial deficiency in the adaptive one. Consequently, this subgroup exhibited innate immune activation and increased mortality [67]. 

The coagulopathic cluster was significant for coagulation disorders, including disseminated intravascular coagulation, which reflected changes in platelet degranulation, glycosaminoglycan linkage, or the coagulation cascade. Moreover, this subgroup exhibited increased mortality. In contrast, the adaptive endotype had greater activation of the adaptive immune genes and interferon signaling, correlating with lower clinical severity and lower mortality [67]. 

Thus, these findings can predict a response for drugs modulating coagulation or the innate or adaptive immune systems in sepsis patients belonging to one of the subtypes [67].

Transcriptomics analysis could explain the variations in the survival benefit linked to the use of corticosteroids in septic shock. Two large clinical trials have examined the influence of corticosteroid use on mortality rates in septic shock [68]. Both revealed that corticosteroids can shorten the duration of shock; however, the influence on survival differed between the two trials, with one of them reporting improved survival [68], while the other reported no difference [69]. To investigate these inconsistencies, Antcliffe et al. examined the interaction between SRS clusters and the response to treatment [70]. Their results showed that patients in the SRS2 immunocompetent group had poorer survival outcomes when treated with corticosteroids than those who were given a placebo [70].

Thus, transcriptomics-based phenotyping in septic patients could be of major importance in future personalized approaches in sepsis. 

## 6. Proteomics

The study of proteomics is based on differentiated protein marker identification in the patient’s serum [71]. These include pro-inflammatory markers, such as IL-6, IL-8, and soluble TNF receptor-1; markers of endothelial injury (angiopoietin-2, intercellular adhesion molecule 1); markers of epithelial injury (surfactant protein-D, sRAGE); and markers of impaired coagulation (plasminogen activator inhibitor 1, protein C).

Calfee et al. identified two phenotypes by examining five cohorts of ARDS patients, hyperinflammatory and hypoinflammatory [72]. The hyperinflammatory phenotype was characterized by amplified inflammatory signals, as evidenced by elevated plasma levels of IL-6, IL-8, and sTNFR-1; lower plasma protein C levels; decreased serum bicarbonate; an increased prevalence of shock; and an increased prevalence of non-pulmonary sepsis, exhibiting lower platelet counts and higher vasopressor use. It was associated with significantly higher mortality and fewer ventilator-free days [72]. 

Conversely, the hypoinflammatory phenotype was characterized by lower levels of IL-6 and IL-8 and higher plasmatic levels of protein C. Moreover, this subgroup revealed a higher survival rate, more ventilator-free days, and more non-pulmonary organ-failure-free days [72]. 

These two phenotypes are responsible for differential mechanical ventilation strategies, regarding the applied positive end-expiratory pressure (PEEP), the fluid management strategy, and simvastatin use. The hyperinflammatory subgroup responded to simvastatin use when compared to the placebo group. This difference in outcomes was not mirrored in the hypoinflammatory subgroup. Various cohort studies have suggested that the two phenotypes previously studied in cohorts of ARDS patients might have prognostic value beyond acute respiratory distress syndrome (ARDS) [73].

Another ARDS phenotyping technique has focused on unbiased clustering methods for plasma biomarker data. Bos et al. measured 20 plasma biomarkers of inflammation, coagulation, and endothelial activation and separated two phenotypes, which they termed “uninflamed” and “reactive” [74]. The severity of hypoxemia as measured by PaO_2_/FiO_2_ was not associated with phenotype membership. The reactive cluster, characterized by higher levels of IL-6, IFN-γ, Ang2/Ang1, and PAI-1, showed significantly worse clinical outcomes in patients with acute hypoxemic respiratory failure, suggesting its prognostic value in this clinical setting. The reactive group was also notable through increased neutrophil activation, oxidative phosphorylation, and mitochondrial dysfunction, compared to the uninflamed group [74]. The precise correspondence between the LCA-defined phenotypes (hyperinflammatory vs. hypoinflammatory) and the reactive versus uninflamed phenotypes should further be studied and could serve as a potential strategy for a personalized approach in sepsis and septic shock. 

## 7. Metabolomics

**Figure 6:**
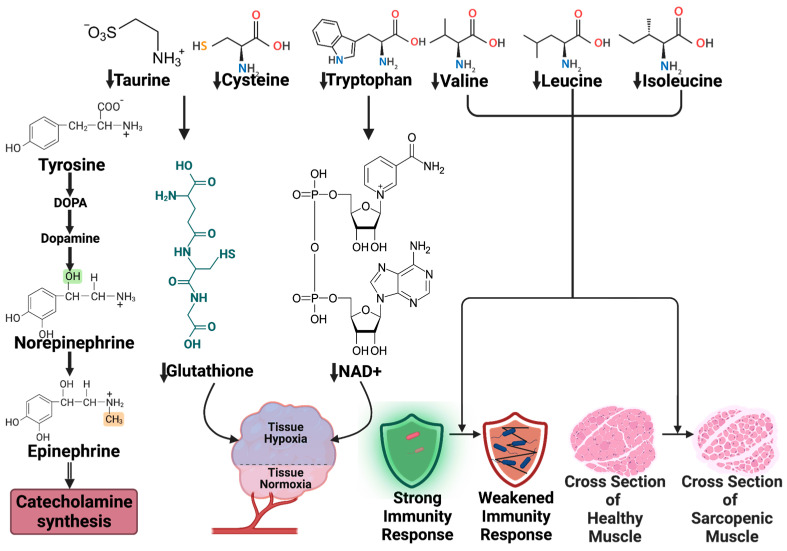
Changes in protein metabolism in septic shock. NAD−nicotinamide adenine dinucleotide.

The study of metabolomics in sepsis has the potential to improve the diagnosis, risk stratification, and treatment of this severe pathophysiological condition. Sepsis is associated with eloquent metabolic changes such as elevated protein catabolism, a negative nitrogen balance, and the modification of amino acid plasma concentrations [75]. The influence of sepsis on protein metabolism is revealed in Figure 6.

Aromatic amino acids include tryptophan, phenylalanine, and tyrosine. In humans, they serve as a precursor for many biologically active molecules. Tyrosine is used for the synthesis of dopamine, noradrenaline, and adrenaline. Tryptophan represents the initial precursor for tryptamine, kynurenine enzymes, and serotonin. Tryptophan levels were shown to decrease in septic patients. The metabolite resulting from these reactions is kynurenine, whose values are increased in sepsis. The enduring depletion of tryptophan results in a drop in NAD+ levels, which will lead to tissue hypoxia [76]. 

Plasmatic levels of taurine and cysteine could be increased in septic patients [77]. However, as the disease progresses, their concentrations start to decline. Cysteine, which is a precursor for glutathione, is a potent antioxidant [77]. Taurine was shown to possess antioxidant effects too [78]. 

Valine, leucine, and isoleucine constitute the branch-chain amino acids (BCAAs) group. Their levels were shown to be decreased in septic patients, correlated to the disease severity. They are involved in muscle protein synthesis, immune function, and the activation of the mTOR pathways. Regardless of the increased insulin resistance and protein catabolism, which are regular features in sepsis and which were found to raise the plasma concentration of valine, the increased catabolism of BCAAs and enhanced activity of the branched-chain alpha-keto acid dehydrogenase (BCKD) complex explain the reduced levels of plasmatic BCAAs. It is widely accepted that BCAAs are essential in muscle protein synthesis, so supplementation in septic patients, especially in those developing sarcopenia, could be an effective tool to reduce mortality and morbidity [79]. 

Lipids are involved in maintaining the balance between the pro-inflammatory and anti-inflammatory processes. A better understanding of the lipid metabolism in sepsis could help to develop novel and more personalized diagnostic and treatment approaches. 

A particular type of lipids, called lipid mediator specialized pro-resolving mediators (LM-SPM), were high in the plasma of septic patients, and these levels were shown to correlate with the evolution to ARDS [80]. Although the LM-SPM values are increased in sepsis, their anti-inflammatory activity is hindered, possibly by the downregulation of its receptors. Prostacyclin levels are elevated in sepsis, this mediator being associated with vasoplegia and cardiovascular dysfunction. Lysophosphatidylcholine (LPC) is a mediator obtained from phosphatidylcholine via the phospholipase A2 pathway. Serum LPC levels are substantially decreased in septic patients. LPC levels increase over time in survivors but remain persistently low in those with severe sepsis, being a strong mortality predictor [80]. 

Platelet-activating factor (PAF) levels are also increased in septic patients. PAF acetylhydrolase, the enzyme involved in PAF metabolism, shows reduced activity in patients with sepsis, this being associated with multiple organ dysfunction. 

HDL presents numerous protective mechanisms in sepsis. However, its levels are low in this patient group. HDL levels are inversely correlated with mortality and organ dysfunction. HDL levels remain low even after the resolution of the disease. Moreover, in sepsis, there is an increase in the number of large HDL molecules, at the expense of small and medium ones. VLDL levels are increased in sepsis, due to the decrease in the ability of the liver to clear them away from the blood [81]. Their main roles remain to be clarified in the future.

Lactic acid is a well-known marker for sepsis. Its elevated level is a very specific mortality predictor and it accumulates as a result of deficient energy metabolism during sepsis [82]. Indeed, increased amounts of substrate (citrate, malate, pyruvate, acetate, lactate) from the tricarboxylic acid cycle are a consistent finding in sepsis patients, especially in non-survivors, due to the inability to metabolize it [83].

The pentose phosphate pathway is employed as a compensatory response to decreased ATP [83]. According to Qiu et al., the pentose phosphate pathway is linked to sepsis-associated encephalopathy [40]. Moreover, Pan et al. found that glycolysis was inhibited in neutrophiles during sepsis, leading to an impaired immune response [84]. 

In conclusion, the analysis of the differentiated metabolic dysregulations in septic patients offers a better explanation for the complexity of sepsis. Thus, it could contribute to a more personalized strategy in this severe clinical condition. 

## 8. Clinical Phenotypes 

**Figure 7:**
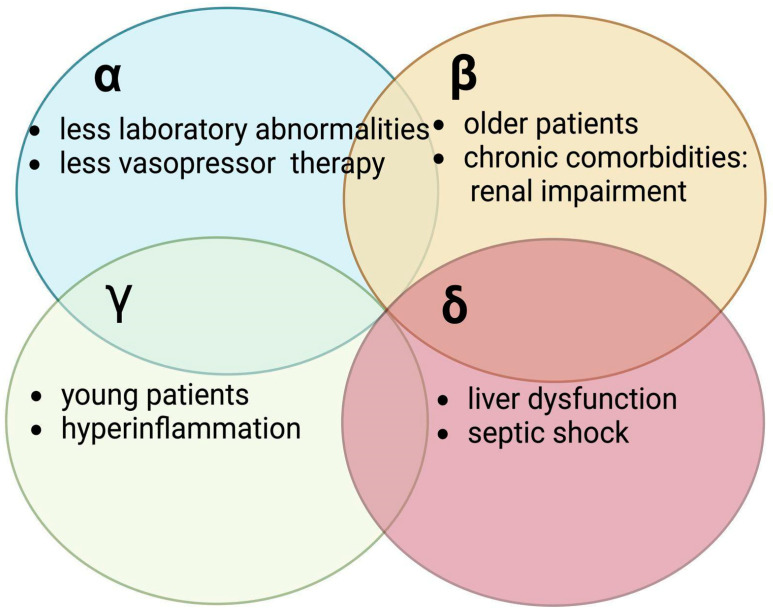
Clinical sepsis phenotypes and their characteristics.

In 2019, Seymour and colleagues performed a retrospective analysis with the aim to identify potential clinical phenotypes in sepsis [85]. Their study analyzed several datasets from observational cohorts and randomized control trials [85]. They identified four clinical sepsis phenotypes: α, β, γ, and δ (Figure 7).

The α phenotype was the most prevalent (one in three patients) and showed the fewest laboratory abnormalities and the lowest need for vasopressor support therapy. Patients in the β phenotype were older and had more associated chronic comorbidities, particularly renal dysfunction. Patients in the γ subgroup were younger and showed the most signs of inflammation. They also had the most frequent pulmonary dysfunction. Other hematologic malignancies could influence, however, the pulmonary response to an infection [86]. δ phenotype patients had more liver dysfunction and were the most likely to develop septic shock [85].

The four phenotypes showed a difference in 28-day mortality: 5% for the α phenotype, 13% for the β phenotype, 24% for the γ phenotype, and 40% for the δ phenotype. Some of the phenotypes also showed resemblances to previously described endotypes derived by analyzing the circulating immune cells [85]. Moreover, the concordance between certain phenotypes and immunologic endotypes could serve as an early approach in the identification of patients who may benefit from immunomodulating therapies.

A 2022 retrospective study by Bhavani et al. aimed to elaborate sepsis sub-phenotypes based on the longitudinal analysis of patients’ vital signs, such as body temperature, cardiac rate, respiratory frequency, and arterial blood pressure [87]. Their main concern was that most prior studies had used static measurements to identify sub-phenotypes, although sepsis is a highly dynamic pathology, with rapidly evolving physiological responses. They identified four different groups with variable characteristics: A, B, C, and D.

Patients from group A were the youngest and their vitals were hyperthermia, tachycardia, tachypnea, and relative hypotension. They had the lowest prevalence of cardiac and renal comorbidities. Group B included patients with hyperthermia, tachycardia, and tachypnea, but associated with hypertension. They had the highest prevalence of cardiac and renal comorbidities. Group C consisted of patients with a lower body temperature and heart and respiratory rate, as well as normal blood pressure. Patients in Group D were the oldest, had a lower temperature and heart and respiratory rate, and were the most hypotensive among the cohort [87].

Groups A and D had the most vasopressor therapy use and significantly higher 30-day mortality. The effect of treatment was also different between sub-phenotypes, with group D having lower odds of mortality when balanced crystalloids were used, compared to use of a saline solution [87].

The novel sub-phenotypes described in the Bhavani study were validated in a temporally distinct validation cohort, thus providing a new perspective for more individualized care for sepsis patients.

A 2021 analysis by Kudo et al. described coagulation phenotypes in sepsis patients [88]. Their cohort consisted of adult patients admitted to the ICU with septic shock. For phenotyping, they measured coagulation markers (platelet count, PT-INR, fibrinogen/fibrin degradation products, D-dimers, and antithrombin activity) after admission. Upon analysis of the data, four clusters were derived: dA, dB, dC, and dD [88].

Patients in the dA cluster had both severe organ dysfunction and severe coagulopathy. Most of these patients required vasopressor therapy. They also had the highest mortality. Patients in cluster dB also presented severe disease, but moderate coagulopathy. Cluster dC contained patients who also presented coagulopathy, but in the context of moderate disease. Patients in cluster dD exhibited mild organ dysfunction and no coagulopathy. Recombinant human thrombomodulin was then administered to patients in all clusters. Patients in cluster dA showed significantly lower 28-day and in-hospital mortality when treated with thrombomodulin. These approaches could be used for the early identification of sepsis patients with severe coagulopathy, which could then benefit from a personalized therapeutic strategy [88].

Approaching septic patients based on clinical features represents a natural first step in the precision-based diagnosis and treatment of sepsis. The advantages of using a clinical personalized approach consist of temporal efficiency and high availability. 

## 9. Conclusions

Observing the complexity of sepsis, the use of new prediction models based on the theory of complex systems using artificial intelligence that include the various sources of heterogeneity should be prioritized. The study of “omics” significantly contributes to this approach. Until the large-scale use of such systems is possible, a personalized phenotype-based strategy seems to be the best tool for the treatment of a septic patient. Regarding the economic aspects, clinical phenotyping represents the most reliable and cost-effective practice in the management of septic patients.

## Data Availability

Data are contained within the article.
